# Dynamic Spectrum Sharing for Future LTE-NR Networks †

**DOI:** 10.3390/s21124215

**Published:** 2021-06-19

**Authors:** Gordana Barb, Florin Alexa, Marius Otesteanu

**Affiliations:** Department of Communications, Politehnica University Timișoara, 300006 Timișoara, Romania; gordana.barb@student.upt.ro (G.B.); marius.otesteanu@upt.ro (M.O.)

**Keywords:** dynamic spectrum sharing, LTE, 5G NR, throughput, spectrum efficiency

## Abstract

5G is the next mobile generation, already being deployed in some countries. It is expected to revolutionize our society, having extremely high target requirements. The use of spectrum is, therefore, tremendously important, as it is a limited and expensive resource. A solution for the spectrum efficiency consists of the use of dynamic spectrum sharing, where an operator can share the spectrum between two different technologies. In this paper, we studied the concept of dynamic spectrum sharing between LTE and 5G New Radio. We presented a solution that allows operators to offer both LTE and New Radio services using the same frequency bands, although in an interleaved mode. We evaluated the performance, in terms of throughput, of a communication system using the dynamic spectrum sharing feature. The results obtained led to the conclusion that using the dynamic spectrum sharing comes with a compromise of a maximum 25% loss on throughput. Nevertheless, the decrease is not that substantial, as the mobile network operator does not need to buy an additional 15 MHz of bandwidth, using the already existing bandwidth of LTE to offer 5G services, leading to cost reduction and an increase in spectrum efficiency.

## 1. Introduction

The next mobile generation following Long Term Evolution (LTE) that has started its deployment in some countries is the 5th Generation (5G). The main focus of LTE was set to increase data transfer rates, while 5G is expected to revolutionize our society, focusing not only on delivering extreme mobile broadband, but also in the fields of critical machine communication and massive machine communication. New applications will emerge, and the target values and requirements proposed are extremely demanding [1]. The main challenges of 5G systems consist of increasing data transfer rates, reducing latency, and increasing capacity, spectrum efficiency, and network energy efficiency, which will be necessary for different application scenarios [2].

The current network architecture cannot sustain all the requirements and target values of 5G New Radio (NR). Therefore, the 3rd Generation Partnership Project (3GPP) released two variations for the new network architecture of 5G NR communication systems: Non-standalone (NSA) and Standalone (SA) [3]. The main difference between both types is that the NSA architecture is based and depends on the LTE core network, while the SA architecture uses a novel next-generation core network, not depending of any LTE infrastructure.

The Internet of Things (IoT), mobile internet, and Cognitive Radio (CR) stand as relevant driving forces for 5G development [4,5,6,7]. The IoT technology has the potential ability to connect almost everything to the internet, which will lead to the massive growth of devices that require network acceptance. Particularly, in 2018, there were approximately 22 billion connected devices, which is the equivalent of around 2.9 devices/person. It is expected that by 2025, the number of connected devices to be nearly 38.6 billion [8]. Simultaneously, the mobile internet has surpassed the traditional mobile communications, offering users innovative experiences. On the other hand, cognitive radio represents a technology that has evolved in order to improve the spectrum usage, exploiting both licensed and unlicensed bands. It became a promising wireless communications technology that is able to solve spectrum problems by: observation, learning, and intelligent adaptation to obtain an optimal frequency band [9].

In addition to the network architecture, another major challenge of 5G systems consists of the available frequency spectrum. Presently, the spectrum used for mobile communications is located between 300 MHz and up to 6 GHz. Due to the fact that most of it is completely saturated, new frequency bands above 6 GHz need to be taken into consideration. Hence, it is expected that 5G NR systems will use frequency bands above 6 GHz, reaching up to 300 GHz [10]. Therefore, the main frequency spectrum to be used for 5G systems can be in licensed, shared, and unlicensed bands, see Table 1. The low-band spectrum has the goal of supporting high coverage and penetration; the mid-band spectrum provides data transfer rates up to 1 Gbps; the high-band spectrum supports peak data transfer rates up to 20 Gbps. The last group, even though it supports such high data rates, comes with the inconvenience of low coverage and penetration, being indicated to be applied in closed/indoor environments.

It is clear that the frequency spectrum is a scarce and limited resource that constitutes an important factor in mobile communication systems, as well as the related cost for the Mobile Network Operator (MNO). In this context, new spectrum explorations [11,12], higher energy efficiency [13], and dynamic spectrum usage [14,15,16,17,18] have become the new features of communication networks.

The topic of spectrum sharing in the bands of old communication systems started drawing the attention of researchers, as it is the safest and most economical solution [19]. The standardization procedure for the spectrum sharing principles started in March 2017 by 3GPP. One of the solutions presented regarding the spectrum allocation for 5G NR systems comprises the use of the existing frequency spectrum used by the already deployed mobile generations.

Spectrum sharing is based on the flexibility of the physical layer and the fact that in the LTE network, all channels are assigned in the time-frequency domain. This way, the flexibility of the 5G NR radio interface can be used for reference signals, allowing dynamic configuration and minimizing collisions between NR and LTE during simultaneous data transmission. Consequently, there is the possibility of sharing a frequency domain within the same communication channel. A comprehensive overview on the different ways of spectrum sharing that has been investigated in recent years is found in [14]. In addition, in [20,21], new schemes and algorithms for dynamic spectrum sharing between Global System for Mobile Communications (GSM) and LTE technologies were investigated. Regarding the IoT, spectrum sharing is a preferable approach to cope with the conflicts between massive IoT connections and limited spectrum resources as it was discussed in [22,23,24,25]. It can also be used to solve vertical requirements and the competition in the acquisition of frequency bands between MNOs [26,27], as well as to improve spectrum utilization in Cognitive Radio (CR) and TV white space [28,29,30]. The leading mobile producers have shown massive interest in developing solutions for dynamic spectrum sharing. These are presented in Table 2.

**Figure 1:**
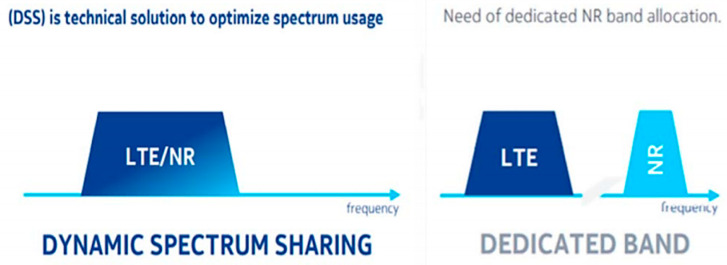
DSS vs. using dedicated bands for NR and LTE [31].

The Dynamic Spectrum Sharing (DSS) solution allows mobile network operators to offer LTE and NR services using the same frequency bands, although in an interleaved mode. This allows NR services without the need of acquiring new and dedicated frequency spectrum, antenna, or radio frequency units. The solution is intended to assist operators in the short-term rollout deployment of 5G services through LTE already-in-use spectrum. It is not intended to provide substantial performance, as that would necessitate new dedicated spectrum for NR, but to provide coverage, reduce costs, and improve spectrum efficiency for the operator. Figure 1 presents the DSS technology with LTE and NR sharing the same frequency band in comparison to using two separate bands for each technology.

The deploying of DSS technology is divided into two phases: Phase 1, which is based only on the NSA architecture and accepts a sharing ratio between 20 and 60% with a fixed UL sharing ratio; and Phase 2, which introduces a dynamic UL sharing ratio and accepts both NSA and SA architectures. The main differences between both phases are presented in Table 3. The sharing ratio refers to the ratio of shared resources between both technologies. For example, considering a sharing ratio of 20% refers to 5G NR occupying 20% of the available resources, while LTE occupies 80%. Another example is for a sharing ratio of 60%, meaning that 5G NR uses 60% of the available resources while LTE uses only 40%.

**Figure 2:**
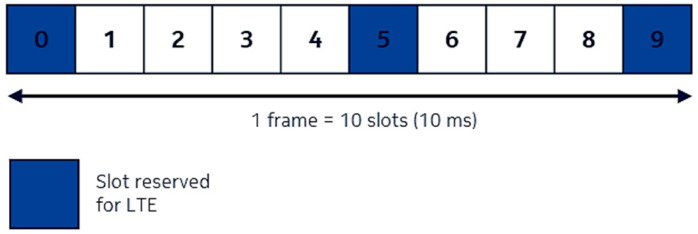
Frame allocation for DL Phase 2 DSS [32].

For downlink, the allocation of the subframes is based on Time Division Multiplexing (TDM). In one frame, regardless of the sharing ratio adopted, subframes 0, 5, and 9 are strictly dedicated to LTE transmission. Subframes 1, 2, 3, 4, 6, 7, and 8 can be used for both LTE and NR transmission, depending on the sharing ratio and the architecture mode adopted, see Figure 2 [32].

**Figure 3:**
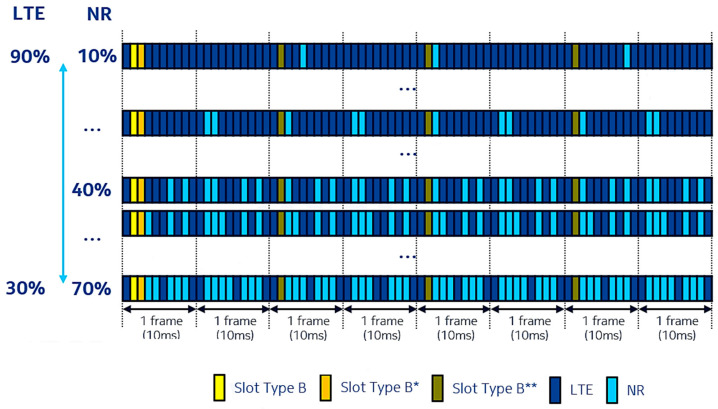
Resource allocation patterns for DL Phase 2 DSS [34].

The downlink resource allocation, when considering the transmission of several frames, varies depending on the sharing ratio implemented [33]. Different patterns are depicted in Figure 3, for the NSA architecture mode. It can be observed that for every frame, subframes 0, 5, and 9 are always dedicated to LTE. In addition, for the first frame only, slot 1 is represented with yellow (slot type B) and slot 2 with orange (slot type B*), and both are used for transmitting synchronization and CSI-RS signals, respectively. Additional synchronization signals are sent with a period of 20 ms in slot 1 of the remaining frames, represented by green (slot type B**). The remaining slots are used for LTE and NR transmission.

**Figure 4:**
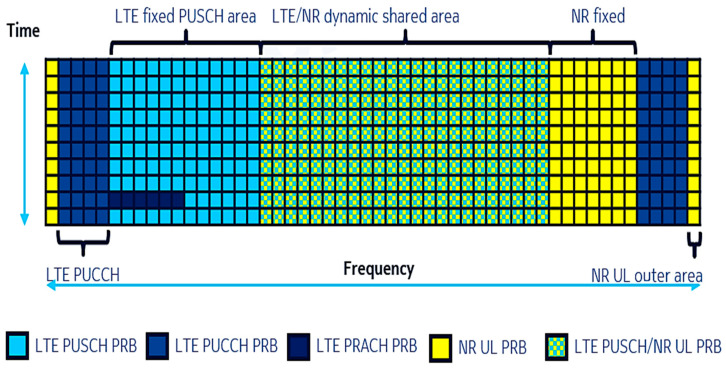
Resource allocation for UL Phase 2 DSS [34].

For uplink, the allocation of the resources is based on Frequency Division Multiplexing (FDM). As depicted in Figure 4, the Physical Resource Blocks (PRB) available for LTE or NR transmission are represented by the color green and depend on the sharing ratio adopted and carrier bandwidth. Furthermore, there are seven PRBs dedicated to NR UL transmission only, represented by the color yellow in the right outer edge of the frequency band. These PRBs depend on the positioning of the LTE PRACH PRBs. In Figure 4, these are located in the left outer edge of the frequency band. If the LTE PRACH PRBs were located in the right outer edge, the seven NR UL PRBs would then be positioned in the left outer edge (meaning the opposite side).

The calculation of the maximum available NR/LTE sharing ratio is given by the following equation:

(1) $${\mathrm{Sharing}\;\mathrm{ratio}}_{\mathrm{Max}\_\mathrm{UL}}=1-\left(\frac{{N+\mathrm{PUCCH}}_{\mathrm{Max}\_\mathrm{LTE}}}{\mathrm{LTE}\;\mathrm{BW}\;\mathrm{in}\;\mathrm{PRBs}}\right)$$

where N represents the number of PRBs that are necessary for LTE transmission, for instance, the LTE PRACH PRBs.

In this paper, we studied the concept of dynamic spectrum sharing between LTE and 5G NR technologies for the same mobile network operator. We assessed the performance of an LTE-NR communication system using the DSS feature, in terms of throughput, using different sharing ratios for both NSA and SA architectures and for both transmission directions (downlink and uplink). We performed a comparison of the performance while using different modulation schemes and numbers of layers. The remainder of the paper is organized as follows. Section 2 presents the sharing ratio calculation for downlink and uplink; Section 3 provides the equipment and methods used for the measurements; and Section 4 presents the results obtained and analysis. Lastly, Section 5 delivers the conclusions of this paper.

## 2. Sharing Ratio Calculation

**Figure 5:**
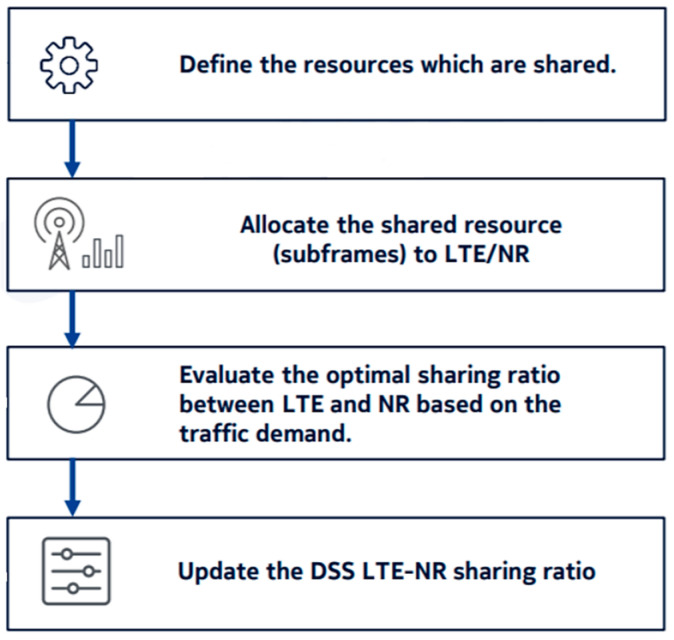
CRM main responsibilities [31].

The sharing ratio between LTE and NR is defined and managed by a new system unit denominated Common Resource Manager (CRM). It has the responsibility to compute the sharing ratio and update it according to traffic demands. In order to do so, the CRM continuously gathers information from both LTE and NR sites. The CRM component is composed by 3 objects: CRM, situated in the base station; LTE CRM, situated in the LTE system unit; NR CRM, situated in the NR system unit. Figure 5 presents the main responsibilities of the CRM. Initially, it starts by gathering information from LTE and NR sites and, based on the information receives, it defines the resources to be shared. It then allocates the shared resources to both technologies. According to traffic conditions and demands for a specific moment, it evaluates the optimal sharing ratio to be selected and finally updates it for LTE and NR.

**Figure 6:**
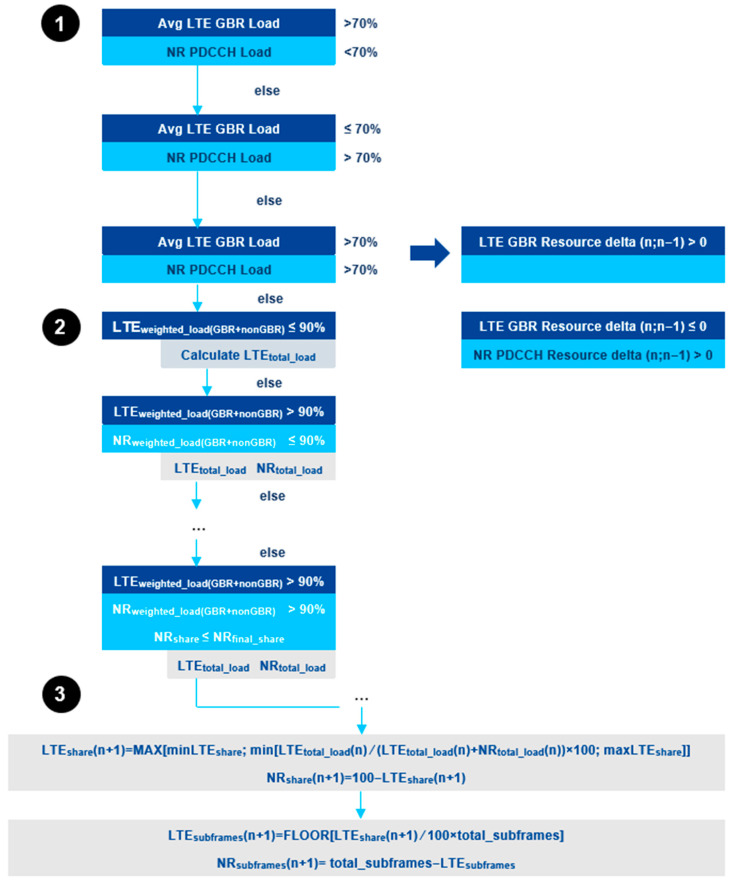
Algorithm for DL Phase 2 DSS sharing ratio calculation [34].

For downlink, depending on the traffic demands for a specific moment, the CRM receives information concerning load indication and takes a decision on the DL sharing ratio that needs to be adopted. To calculate the DL load, the weighted load (based on PRB occupancy), average LTE DSS Guaranteed Bit Rate (GBR) load, NR DSS GBR load, and NR PDCCH load need to be determined [35]. The algorithm for the sharing ratio calculation for DL is presented in Figure 6. The first step consists of the verification, from the CRM entity, of the average LTE GBR load, as well as the NR PDCCH load against a defined threshold, so that a decision can be taken regarding the resources to be assigned. If the average LTE GBR load is higher than 70% and the NR PDCCH load is lower than 70%, then the sharing ratio for NR will decrease. Else, if the average LTE GBR load is equal or lower than 70% and the NR PDCCH load is higher than 70%, then the sharing ratio for NR will increase. Lastly, if both the average LTE GBR load and NR PDCCH load are higher than 70%, then one of the two following conditions is applied:

- If the LTE GBR resource delta (*n*; *n* − 1) > 0, the sharing ratio for NR will be reduced;
- If the LTE GBR resource delta (*n*; *n* − 1) ≤ 0 and the NR PDCCH resource delta (*n*; *n* − 1) > 0, the sharing ratio for NR will increase.

The second step of the algorithm is based on the load information received from step 1. The CRM then calculates the LTE-weighted load and the NR weighted load, from which the LTE and NR total loads are determined. Finally, in step 3, the number of LTE and NR subframes is calculated, taking into account the LTE and NR total loads from step 2. The resulting number of subframes matches to a specific sharing ratio value.

**Figure 7:**
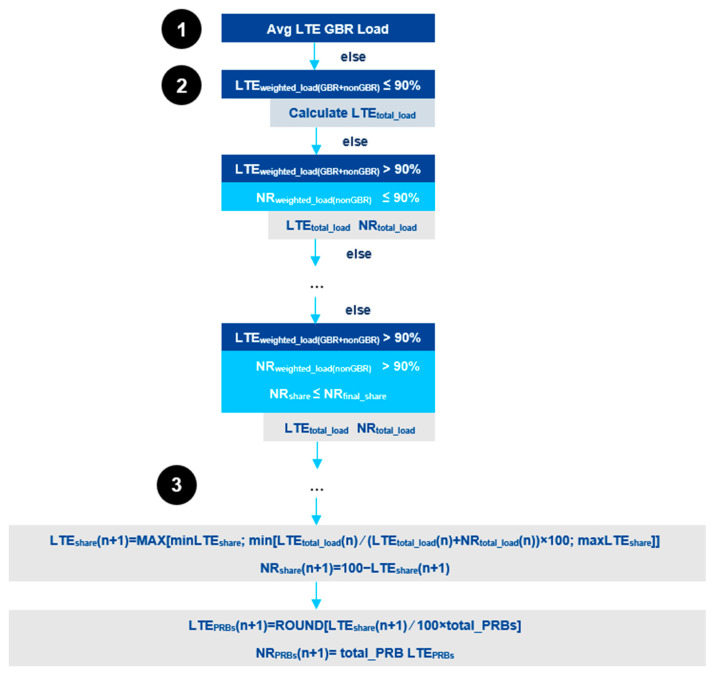
Algorithm for UL Phase 2 DSS sharing ratio calculation [34].

For uplink, a similar procedure as for downlink is taken. Depending on the traffic demands for a specific moment, the CRM receives information concerning load indication and takes a decision on the UL sharing ratio that needs to be adopted. To calculate the UL load, the weighted load (based on PRB occupancy) and the average LTE DSS GBR load need to be determined. Figure 7 presents the algorithm for the UL sharing ratio calculation. For step 1, the average LTE GBR load is verified by the CRM, so a decision can be taken regarding the assignment of resources. If the average LTE GBR load is higher than 70%, then the sharing ratio for NR will be decreased. The second step of the algorithm is based on the load information received from step 1. The CRM then calculates the LTE weighted load and the NR weighted load, from which the LTE and NR total loads are determined. Finally, in step 3, the number of LTE and NR subframes is calculated, taking into account the LTE and NR total loads from step 2. The resulting number of subframes matches to a specific sharing ratio value.

## 3. Equipment and Methods

This section presents the parameters adopted for our work, as well as the scenarios tested. We considered a MIMO system, composed of one base station for LTE, one for NR, and one mobile station that is composed of a Qualcomm chipset prototype that is frequently used on commercial Samsung devices. We used both 64QAM and 256QAM modulation for the measurements. A bandwidth of 15 MHz was selected. The Absolute Radio-Frequency Channel Numbers (ARFCN) for NR were 175,800 for downlink and 166,800 for uplink. The NR-ARFCN is a code that refers to the carrier frequency to be used for both transmission directions of the radio channel and is defined in the 3GPP TS 38.104 Release 16 specification [36]. The NR-ARFCN can be converted to frequency, resulting in 75,800 = 879 MHz for downlink and 166,800 = 834 MHz for uplink. Frequency Division Duplex (FDD) was selected for all cases. We performed throughput measurements using physical and static equipment from Nokia Networks R&D laboratory, considering a Signal-to-Interference-plus-Noise Ratio (SINR) higher than 25 dB and a Reference Signal Receive Power (RSRP) higher than −70 dBm with Line of Sight (LoS) and without the presence of fading. These are standard values used at the laboratory for testing the performance of new technologies. They are considered very good radio conditions, and the reason for choosing them is to create almost ideal radio conditions in order to verify and confirm the aptness of DSS technology and its peak performance using physical measurements, as it is a technology under development and testing. We considered both NSA and SA architectures. For NSA, we measured using sharing ratio values between 20 and 70%. For SA, we measured using sharing ratio values between 30 and 70%. Table 4 below summarizes the scenario parameters adopted for the work.

**Figure 8:**
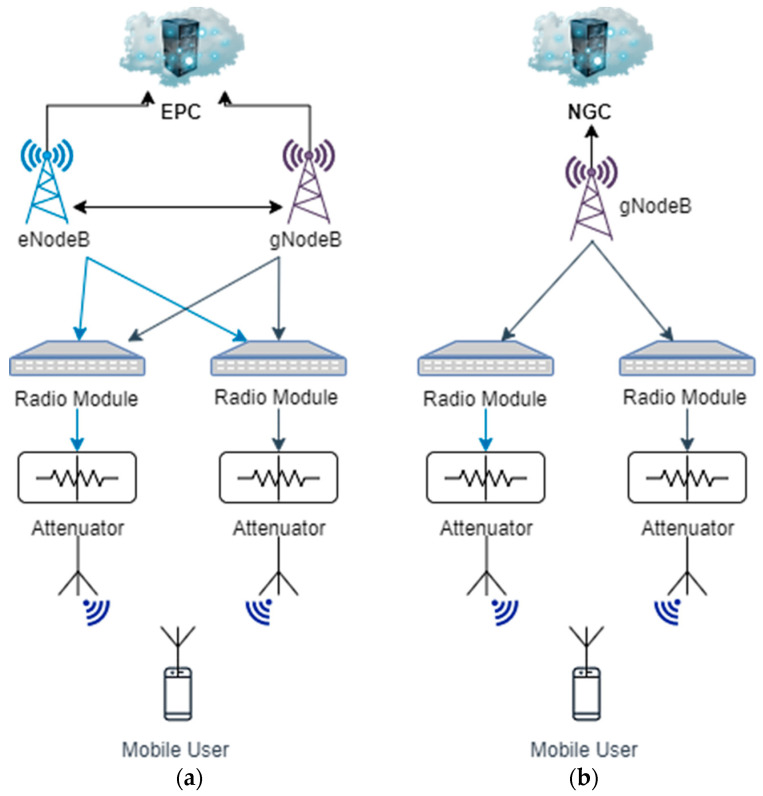
Architecture diagram adopted for the measurements: (**a**) NSA architecture; (**b**) SA architecture.

Each network architecture type has different possible variations. The NSA architecture is based on the LTE core network and uses LTE-based interfaces. For this type of architecture, the gNodeB needs to support these interfaces and acts as a secondary node, while the eNodeB acts as a primary or master node. There are different options to deploy an NSA architecture—option 3, 3a, 3x, 4, 4a, 7, and 7a. The option used for our measurements is NSA option 3x, where the control plane is routed through the master eNodeB and the user plane is directly routed through the secondary gNodeB. The eNodeB also communicates directly with the gNodeB and both communicate directly with the Evolved Packet Core (EPC). The SA architecture has 2 options—option 2 and 5. Option 2 is that adopted for our work and, as it can be seen in Figure 8b, consists of a Next Generation Core (NGC) and a gNodeB that communicates directly with it, without needing any support of LTE structures. For both network architectures studied, we used two radio modules that have attached to them one attenuator, as the measurements were performed in a laboratory with close proximity to the mobile user. We used either 2 or 4 antennas, depending on the case studied.

## 4. Results and Discussion

This section presents the results obtained from our measurements. We divide it into two subsections: downlink and uplink results. In each subsection, we present results for both NSA and SA architectures. We considered sharing ratios ranging from 20 to 70% for the NSA architecture and from 30 to 70% for the SA architecture, meaning that 5G NR occupies between 20 and 70%, and 30 and 70% of the available resources, while LTE occupies between 80 and 30%, and 70 and 30% for the NSA and SA architectures, respectively.

### 4.1. Downlink

**Figure 9:**
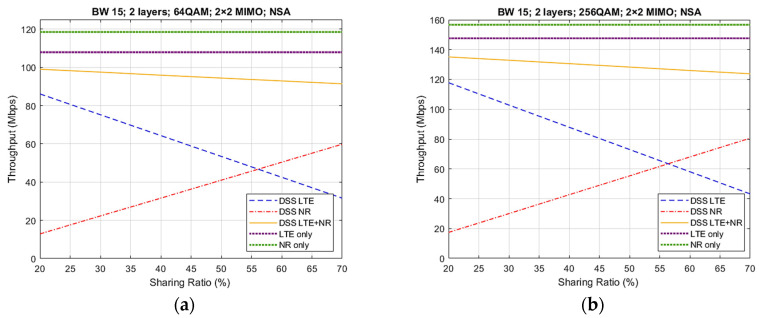

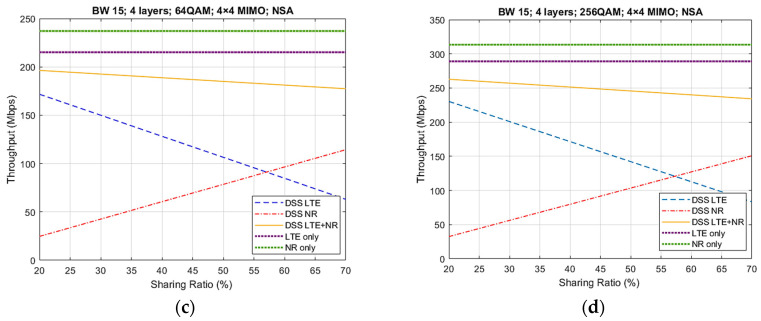
DL throughput results for NSA architecture, with sharing ratios from 20% up to 70%: (**a**) Case 1 results; (**b**) Case 2 results; (**c**) Case 3 results; (**d**) Case 4 results.

Figure 9 presents the throughput results using the DSS feature from the first four cases of Table 2, comprising the NSA architecture. The differences between the cases consist of the modulation type, that is either 64QAM or 256QAM modulation, and MIMO type, that is either 2 × 2 or 4 × 4 MIMO. Each case depicts five different curves. The green (NR only) and purple (LTE only) curves represent the values for the throughput achieved without the use of DSS technology. The yellow curve (DSS LTE + NR) is the most important one as it presents the total throughput obtained with DSS. The remaining blue (DSS LTE) and red (DSS NR) curves represent the individual throughputs for each technology while using DSS. Notice that the DSS LTE + NR throughput equals the sum of the individual DSS LTE and DSS NR throughputs. It can be observed that, for all cases, when we increased the sharing ratio, the values for the DSS NR TP also increased while the DSS LTE TP values decreased. This was an expected behavior, as the higher the sharing ratio, the more resources will be available for NR transmission and the fewer for LTE transmission. It can also be observed that from a sharing ratio of approximately 57%, meaning that NR occupies 57% of the resources while LTE occupies 43%, the individual throughput for NR surpassed the LTE throughput. In addition, it is visible that the overall throughput using DSS was slightly lower than that for the LTE or NR-only throughputs. This is understandable, as the available frame resources are shared between both technologies.

Comparing case 1 and case 2, where the difference is the modulation type that increases from 64QAM to 256QAM modulation, it can be concluded that the major difference is on the maximum values for throughput. For case 1, depending on the sharing ratio adopted, between 90 and 100 Mbps were obtained, while for case 2, the values for throughput were approximately 120–135 Mbps. An increase of 35% was observed. A similar comparison can be conducted for cases 3 and 4. For case 3, the maximum DSS throughput values were 175–200 Mbps, while for case 4, the values varied between 240 and 260 Mbps, depending on the sharing ratio. For these cases, an increase of 37% in throughput was observed for a sharing ratio of 20%, while for a 70% sharing ratio, the throughput increase was 30% (see Table 5).

Regarding case 1 and case 3, where the difference consists of the number of transmitting and receiving antennas, 2 × 2 MIMO and 4 × 4 MIMO, respectively, an increase in all throughput values of approximately 50% could be observed, along with an increase in complexity.

**Figure 10:**
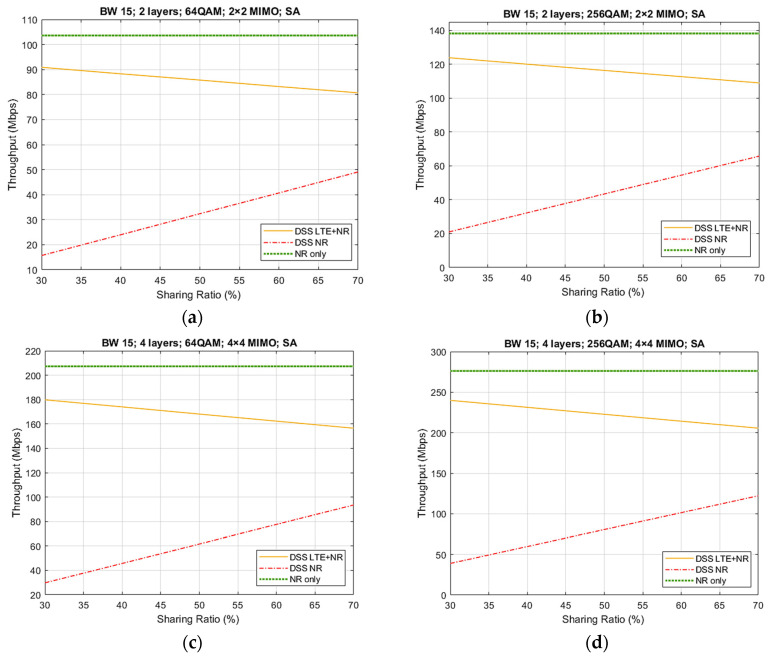
DL throughput results for SA architecture, with sharing ratios from 30% up to 70%: (**a**) Case 1 results; (**b**) Case 2 results; (**c**) Case 3 results; (**d**) Case 4 results.

Figure 10 depicts the DL throughput results with the DSS feature for the first four cases from Table 2, using the SA architecture, contrary to the results from Figure 8 that made use of the NSA architecture. The main difference of both types of architectures is that NSA is an intermediary solution that is based on the LTE network, while the SA architecture does not depend in any way on the LTE network, using a Next-Generation Core (NGC) along with NR protocols. Moreover, the SA architecture leads to an improved efficiency with less complexity. Comparing case 1 and case 2, we can observe that maximum DSS throughput values varied between 80 and 90 Mbps, and 110 and 120 Mbps, respectively. The increase from one case to another was approximately 36%. For cases 3 and 4, the DSS values varied between 160 and 180 Mbps, and 200 and 240 Mbps, respectively. For these, an increase of approximately 29% was observed.

Regarding case 2 and case 4, the increase in the DSS throughput was approximately 87% for both sharing ratios of 30% and 70%. In addition, it can be remarked that for all cases, the values for the NR-only throughput for the SA architecture were smaller than those of the NSA architecture, with a difference of around 15 Mbps for case 1, 20 Mbps for cases 2 and 3, and 40 Mbps for case 4. The reason for this is that in the SA architecture, the number of broadcast signals was higher than that in the NSA architecture. An example is the presence of System Information Block (SIB) signals, as well as paging with information regarding the cell.

Table 6 provides the percentage loss of the throughput that occurs when using DSS instead of an NR-only system. It can be observed that there was a loss in throughput values between a minimum of 10% and a maximum of 26%, depending on the sharing ratio and case adopted. The existence of a decrease in throughput was expected, as with DSS, the available resources are shared with LTE, and hence, there are fewer resources available for NR, compared to a system that is based only on NR. However, from the results obtained, for the NSA architecture, having a loss between 14 and 25% is not a considerable decrease taking in account the fact that there is no need for new dedicated spectrum to be allocated for NR, as it shares the bandwidth with LTE technology. For case 1, the average loss was 19.8% for NSA and 17.2% for SA. For case 2, the loss was 17.5% for NSA and 15.6% for SA. For case 3, we had a 21.2% loss for NSA and 19% for SA. Lastly, for case 4, the loss was 20.6% for NSA and 19.2% for SA.

We can observe that the decrease in the DSS throughput was higher with the increase in the sharing ratio. This is due to the fact that, as the sharing ratio increases (meaning that a higher number of the available resources were used for NR transmission and fewer for LTE), more synchronization signals are transmitted in the slots dedicated to NR, as well as overhead signals.

### 4.2. Uplink

**Figure 11:**
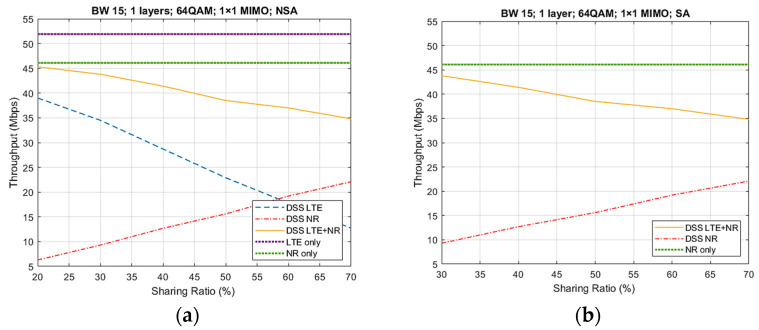
UL throughput results for case 5. (**a**) NSA architecture; (**b**) SA architecture.

Figure 11 depicts the UL throughput results for case 5 from Table 6, using both NSA and SA architecture. Regarding the NSA architecture, it can be observed that the maximum throughput values for LTE and NR only (not considering the DSS feature) were 40 and 22 Mbps, respectively. Moreover, if we compare the results of the DSS LTE + NR and NR-only throughputs from both architecture types, we can conclude that they were similar. Therefore, there is no difference between the SA and NSA architecture, as there are no additional channels that need to be transmitted and hence occupy extra resources.

Table 7 presents the percentage loss of throughput when using the DSS technology. We can observe that for both architecture types, a maximum loss of 25% occurred for a sharing ratio of 70%, meaning that NR occupied 70% of the available resources while LTE occupied only 30%. Therefore, we can conclude that using the DSS technology comes with a compromise of a maximum 25% loss on throughput. However, the decrease observed is not that considerable if taking into account that, instead of needing an additional 15 MHz of bandwidth, the system shares the actual 15 MHz between both technologies. Subsequently, from the operator’s point of view, DSS brings the advantage of cost reduction and spectrum efficiency, while being able to present the “5G icon game” and provide coverage strategies.

## 5. Conclusions

In this paper, we analyzed the impact and the advantages of using the DSS technology in an LTE-NR communication system. We proposed different schemes for the resource allocation, according to the selected sharing ratio. The results obtained provided insight into the behavior of the system with DSS and showed that it is a technology that brings advantages from the operator’s point of view, mainly regarding the spectrum efficiency and cost reduction. In conclusion, from the results obtained, it is clear that using the DSS technology brings a major advantage of not needing extra dedicated bandwidth for NR systems, which, from the operator’s point of view, leads to an improvement of spectrum efficiency and cost reduction. We performed a comparison of the spectrum usage for LTE and NR when adopting the DSS feature and without. In addition, we measured the throughput obtained for both LTE and NR, using the proposed allocation schemes for each sharing ratio. The results obtained clearly demonstrated that even if a maximum loss of 25% on throughput is observed, there is a major advantage in using the DSS technology due to the fact that there is a cost reduction for the mobile operator alongside an optimization on the spectrum usage, due to the fact that the MNO can re-use the already existing 15 MHz bandwidth of LTE and does not need to buy any new dedicated supplementary 15 MHz for 5G services.

In conclusion, the deployment of DSS technology is useful, especially for the initial deployment of 5G NR, as the operator is able to build strategies while presenting the initial 5G picture to the consumer, through LTE already-in-use spectrum.

## Figures and Tables

**Table 1:** 5G spectrum options.

Frequency Band	Characteristics	Spectrum Use
600–2600 MHz	Wide area coverage	Spectrum sharing
	Reframing from old technologies	
3.3–5 GHz	Mid-band mainstream for 5G	Independent deployment (without old technologies)
	Massive MIMO-crowded and urban areas	
5–6 GHz	Unlicensed spectrum	
	No spectrum licensed necessary	
24–39 GHz	Allocated mmWaves for 5G	
	Increased data rates	

**Table 2:** Leading producers and adopted DSS solution.

Leading Producers	Name of DSS Solution
**Ericsson**	ESS—Ericsson Spectrum Sharing
**Huawei**	CloudAIR
**MediaTeck**	Dimensity 1000
**Nokia**	Dynamic Spectrum Sharing (DSS)
**QualComm**	Snapdragon X60
**Samsung**	DSS
**ZTE**	SuperDSS/Magic Radio Pro

**Table 3:** Comparisons between two phases of DSS implementation.

Phase 1	Phase 2
**Sharing ratio 20–60%**	Sharing ratio 5–70%
**Fixed UL sharing ratio**	Dynamic UL sharing ratio
**Only NSA architecture**	NSA and SA architectures
**Supports only FDD**	Supports FDD and TDD
**Sharing ratio update is slow**	Sharing ratio update takes up to 100 ms

**Table 4:** Set of parameters adopted for our work.

Case	Direction	Bandwidth	Nr. of Layers	Modulation	MIMO
1	DL	15	2	64QAM	2 × 2
2	DL	15	2	256QAM	2 × 2
3	DL	15	4	64QAM	4 × 4
4	DL	15	4	256QAM	4 × 4
5	UL	15	1	64QAM	1 × 1

**Table 5:** DL throughput results for DSS LTE + NR in Mbps.

	DL NSA DSS LTE + NR (Mbps)					DL SA DSS LTE + NR (Mbps)				
	30%	40%	50%	60%	70%	30%	40%	50%	60%	70%
**Case 1**	97.5	95.9	94.4	92.9	91.4	90.9	88.3	85.8	83.2	80.7
**Case 2**	132.9	130.6	128.3	126	123.8	123.9	120.1	116.4	112.7	109
**Case 3**	192.6	188.8	185	181.2	177.4	179.8	174	168.1	162.3	156.5
**Case 4**	257.1	251.4	245.7	240	234	239.9	231.3	222.7	214.2	205.6

**Table 6:** Loss of DL throughput, in %, of the DSS LTE + NR value in comparison to NR only.

Sharing Ratio	20%	30%	40%	50%	60%	70%	30%	40%	50%	60%	70%
	NSA ARCHITECTURE						SA ARCHITECTURE				
**Case 1**	−17%	−18%	−19%	−20%	−22%	−23%	−12%	−15%	−17%	−20%	−22%
**Case 2**	−14%	−15%	−17%	−18%	−20%	−21%	−10%	−13%	−16%	−18%	−21%
**Case 3**	−17%	−19%	−20%	−22%	−24%	−25%	−13%	−16%	−19%	−22%	−25%
**Case 4**	−16%	−18%	−20%	−22%	−23%	−25%	−13%	−16%	−19%	−22%	−26%

**Table 7:** Loss of UL throughput, in %, of the DSS LTE + NR value in comparison to NR only.

Sharing Ratio	20%	30%	40%	50%	60%	70%	30%	40%	50%	60%	70%
	NSA ARCHITECTURE						SA ARCHITECTURE				
**Case 5**	−2%	−5%	−10%	−17%	−20%	−25%	−5%	−10%	−17%	−20%	−25%

## References

[1] Shafi M., Molisch A.F., Smith P.J., Haustein T., Zhu P., De Silva P., Tufvesson F., Benjebbour A., Wunder G. (2017). 5G: A Tutorial Overview of Standards, Trials, Challenges, Deployment, and Practice. IEEE J. Sel. Areas Commun..

[2] Adebusola J.A., Ariyo A.A., Elisha O.A., Olubunmi A.M., Julius O.O. An Overview of 5G Technology. Proceedings of the 2020 International Conference in Mathematics, Computer Engineering and Computer Science (ICMCECS).

[3] Rhayour A.E., Mazri T. 5G Architecture: Deployment scenarios and options. Proceedings of the 2019 International Symposium on Advanced Electrical and Communication Technologies (ISAECT).

[4] Agiwal M., Roy A., Saxena N. (2016). Next generation 5G wireless networks: A comprehensive survey. IEEE Commun. Surv. Tutor..

[5] Dymkova S.S. (2019). Breakthrough 5G data call using dynamic spectrum sharing to accelerate nationwide 5G deployments. Synchroinfo J..

[6] 5G NR and 4G LTE Coexistence: A Comprehensive Deployment Guide to Dynamic Spectrum Sharing [Online]. https://newsletter.mediatek.com/hubfs/mediatek5gprogress/Dynamic-Spectrum-Sharing-WhitePaper-PDFDSSWP-031320.pdf.

[7] Tikhvinskiy V., Deviatkin E., Aitmagambetov A., Kulakaeva A. Provision of IoT Services for CO-Located 4G/5G Networks Utilization with Dynamic Frequency Sharing. Proceedings of the 2020 International Conference on Engineering Management of Communication and Technology (EMCTECH).

[8] Number of IoT Connected Devices Worldwide in 2018, 2025 and 2030 [Online]. https://www.statista.com/statistics/802690/worldwide-connected-devices-by-access-technology/.

[9] Pandit S., Singh G. (2017). An overview of spectrum sharing techniques in cognitive radio communication system. Wirel. Netw..

[10] Matinmikko-Blue M., Yrjölä S., Seppänen V., Ahokangas P., Hämmäinen H., Latva-aho M. Analysis of Spectrum Valuation Approaches: The Viewpoint of Local 5G Networks in Shared Spectrum Bands. Proceedings of the 2018 IEEE International Symposium on Dynamic Spectrum Access Networks (DySPAN).

[11] Li S., Da Xu L., Zhao S. (2015). The internet of things: A survey. Inf. Syst. Front..

[12] Ning H., Liu H., Ma J., Yang L.T., Wan Y., Ye X., Huang R. (2015). From Internet to Smart World. IEEE Access.

[13] Chilipirea C., Ursache A., Popa D.O., Pop F. Energy efficiency and robustness for IoT: Building a smart home security system. Proceedings of the (ICCP).

[14] Tehrani R.H., Vahid S., Triantafyllopoulou D., Lee H., Moessner K. (2016). Licensed Spectrum Sharing Schemes for Mobile Operators: A Survey and Outlook. IEEE Commun. Surv. Tutor..

[15] Zhang L., Xiao M., Wu G., Alam M., Liang Y.-C., Li S. (2017). A survey of advanced techniques for spectrum sharing in 5G networks. IEEE Wirel. Commun..

[16] Gandotra P., Jha R.K., Jain S. (2017). Green communication in next generation cellular networks: A survey. IEEE Access.

[17] Massaro M., Beltrán F. (2020). Will 5G lead to more spectrum sharing? Discussing recent developments of the LSA and the CBRS spectrum sharing frameworks. Telecommun. Policy.

[18] Jorswieck E.A., Badia L., Fahldieck T., Karipidis E., Luo J. (2014). Spectrum sharing improves the network efficiency for cellular operators. IEEE Commun. Mag..

[19] Yu G., Li G.Y., Wang L., Maaref A., Lee J., Lopez-Perez D. (2016). Guest Editorial: LTE in Unlicensed Spectrum. IEEE Wirel. Commun..

[20] Guohua Z., Tianle D., Li Y. A dynamic spectrum re-allocation scheme in GSM and LTE co-existed networks. Proceedings of the 2014 International Symposium on Wireless Personal Multimedia Communications (WPMC).

[21] Jo M., Klymash M., Maksymyuk T., Kozlovskiy R. Dynamic spectrum sharing algorithm for combined mobile networks. Proceedings of the 2014 20th International Conference on Microwaves, Radar and Wireless Communications (MIKON).

[22] Irnich T., Kronander J., Selén Y., Li G. Spectrum sharing scenarios and resulting technical requirements for 5G systems. Proceedings of the 2013 IEEE 24th International Symposium on Personal, Indoor and Mobile Radio Communications (PIMRC Workshops).

[23] Whitmore A., Agarwal A., Xu L.D. (2015). The Internet of Things. A survey of topics and trends. Inf. Syst. Front..

[24] Palattella M.R., Dohler M., Grieco A., Rizzo G., Torsner J., Engel T., Ladid L. (2016). Internet of Things in the 5G Era: Enablers, Architecture, and Business Models. IEEE J. Sel. Areas Commun..

[25] Zhang L., Liang Y., Xiao M. (2019). Spectrum Sharing for Internet of Things: A Survey. IEEE Wirel. Commun..

[26] Lin S., Kong L., Gao Q., Khan M.K., Zhong Z., Jin X., Zeng P. (2017). Advanced Dynamic Channel Access Strategy in Spectrum Sharing 5G Systems. IEEE Wirel. Commun..

[27] Andrews J.G., Buzzi S., Choi W., Hanly S.V., Lozano A., Soong A.C., Zhang J.C. (2014). What Will 5G Be?. IEEE J. Sel. Areas Commun..

[28] Bogucka H., Kryszkiewicz P., Kliks A. (2015). Dynamic spectrum aggregation for future 5G communications. IEEE Commun. Mag..

[29] Yang Y., Dai L., Li J., Mumtaz S., Rodriguez J. (2017). Optimal Spectrum Access and Power Control of Secondary Users in Cognitive Radio Networks. EURASIP J. Wirel. Commun. Netw..

[30] Hu F., Chen B., Zhu K. (2018). Full Spectrum Sharing in Cognitive Radio Networks toward 5G: A Survey. IEEE Access.

[31] Nokia Networks (2020). LTE-NR DSS Phase I.

[32] Barb G., Otesteanu M., Roman M. Dynamic Spectrum Sharing for LTE-NR Downlink MIMO Systems. Proceedings of the 2020 International Symposium on Electronics and Telecommunications (ISETC).

[33] Bhattarai S., Park J.J., Gao B., Bian K., Lehr W. (2016). An overview of dynamic spectrum sharing: Ongoing initiatives, challenges, and a roadmap for future research. IEEE Trans. Cogn. Commun. Netw..

[34] Nokia Networks (2020). LTE-NR DSS with CRS Rate Matching.

[35] Kliks A., Kryszkiewicz P. (2017). Multichannel simultaneous uplink and downlink transmission scheme for flexible duplexing. J. Wirel. Com. Netw..

[36] 5G; NR; Base Station (BS) Radio Transmission and Reception. https://www.etsi.org/deliver/etsi_ts/138100_138199/138104/16.07.00_60/ts_138104v160700p.pdf.

